# Racial Science and Modern Politics

**Published:** 1891-05-23

**Authors:** 


### THE DOCTOR'S ARMCHAIR.

### RACIAL SCIENCE AND MODERN POLITICS.

THE modern Jew is not held by modern scientists to be a very scientific person: but the Jewish race is historically one of the most scientific of all races. The striking thing about the sanitary code of Moses is not its antiquity, nor its inclusiveness, nor even its soundness, but its universal and practical acceptance by every individual of the Hebrew race. Many Jews practice the sanitary parts of their religion intelligently, many blindly; but whether intelligently or blindly they do actually and persistently practice them. The Jew is blamed for his tenacity; he ought to be praised for it. Tenacity of purpose is one of the most effective of all human endowments. Without it no race and no individual can be great: with it races the most defective in some other high human attributes may play a most conspicuous and enduring part in human affairs.

The Jews are disliked by some in all countries, and by all in some countries. Russia, at any rate until lately, had made up her mind to sweep them out root and branch. Before such a resolution could have been arrived at by a whole nation the Jews must have been the occasion of many irritating practical discomforts to their Russian neighbours. It is inconceivable that fanaticism alone should so simultaneously and powerfully animate a whole people as to cause them to expel by millions their fellow countrymen of another race. An explanation is rather to be sought in the peculiar characteristics of the Jews themselves. This, at any rate, accords better with the facts of universal history, and also with the natural indolence and indifference to mere ideas which science has taught us is the peculiarity of all sorts of human beings in the mass.

An ordinary scientist, that is a scientist without genius, considers that a minute attention to details is the scientific method *par excellence*. But the statesman, who has the ambition of scientific statesmanship, must not only consider things in their units, but also in their universals. Whilst it is true that if you take care of the pence the pounds will take care of themselves, it is equally true that those pounds which thus devote themselves to self custody will be exceedingly few in number. The Russian politicians ought to give themselves up at the present time to the consideration of the citizen-value of the Jews as compared with the citizen-value of the native Russians and other pure races. In order to arrive at a just estimate of the comparative citizen-value of the Hebrew race as compared with other races, it is necessary, above all things, to overlook mere minute details and to take a historical survey of the philosophical ideas, practical scientific attainments, and power of survival and persistence displayed by the Jewish people.

There are three lines of investigation which may be pursued in this connection. It may be inquired first—What has been the comparative philosophical or intellectual capacity of Jewish thinkers? Secondly, what has been the standard of practical morals to which the best Jewish moralists have attained? And, thirdly, what has been the nature and extent of Jewish sanitary progress? If it can be shown that in these leading particulars the Jewish race has from a remote antiquity occupied a leading position, then it must be held that the citizen-value of the race is very high, and that any nation or body of statesmen which is determined at all risks to root out such a race in favour of a race less well endowed, is like a man who, having his pockets filled with silver and bronze money, deliberately throws the silver away and retains the bronze.

Among purely speculative thinkers the Jewish philosophers are not held of much account; but among practical thinkers, like the English, their place is very high. We Britons hold that he who reduces a practically infinite number of phenomena to unity, and to a unity so philosophical as to be unassailable, is a first rate philosopher. This is what the Jew has done, and done thousands of years ago. From matter he ascended to minds; from minds many and apparently infinitely diverse he advanced to minds few and less diverse; until finally he reached forward to the grand conception of one mind, one reason, one purpose, one creative energy, and one persistent and all-pervading will. This he named "Being": "I am." When Moses asked the presence in the bush "What is thy name?" the answer was startling in its philosophical originality and in its bold completeness. "Say to the children of Israel—'I AM' hath sent me unto you." No process of deliberately conscious reasoning seems to have preceded or followed this wonderful result of induction or of revelation. The practical Jew, like the practical Englishman, saw, felt, and possessed himself of the truth. From that day to this the leaders of the race have held to the great fundamental idea of the unity of the divine mind. To the Jew, and not to the Greek, or the Confucian, the world owes this philosophically grand and profoundly utilitarian affirmation of intellectual unity. The beginning of all things is mind, and one mind. God is one, and never can be more.

The standard of practical morals attained by the Hebrew race in the earlier periods of its history, and retained until now, is one of the very highest the world has ever seen. What is the characteristic note of the noblest Hebrew literature, the Old Testament? It is justice, practical justice; justice as a sentiment, but justice also in the common details of life. Says Isaiah in a splendid outburst of kingly indignation—"Why do you bring your sacrifices to me? Do I need them? Am I pleased with them? Take them away, and give your minds to plain duties: cease to do evil, learn to do well; devote yourselves to the study of the art of right living; seek judgment, relieve the oppressed, judge the fatherless, plead for the widow." Will anyone who is familiar with the inherent philosophy and practical sense of the Old Testament deny that this note of justice between man and man, of righteousness of conduct, is the most marked characteristic of Jewish morality as expressed in Jewish literature? But a race that has once risen to such a standard, by whatever means; and that still avowedly lives, however imperfectly, by such a standard and no other, is a race which demands quite different treatment at the hands of the scientific statesman from that of final and irrevocable rejection and extirpation.

The standard of sanitation reached by the Jews, and reached by them when some of the most prominent modern nations not only had neither science nor sanitation, but hardly had an existence—this standard is so familiarly known to all intelligent persons that it scarcely needs to be discussed. It is quite apparent to every thinking man that in philosophy, in practical morals, and in physical science, the Jew has a literature and a history, noble and almost unique. It is true he is a keen man of business; it is true that he sometimes gets the better of his western competitor in a variety of ways. But it is not true that the average Jew is less honest than the average Western, or less loyal to conscience and to duty. Western nations have more to gain than to lose by giving the Jew a home and equality amongst them: if racial science and racial history teach anything at all it is this. Russia, by insisting upon the universal expulsion of the Jews, plays the part of an irresponsible madman.

